# The Publishers of the American Journal of Dental Science

**Published:** 1881-03

**Authors:** 


*The Publishers of this Journal*, and the editors, have jogged along now for a good many years quietly and steadily, without jar or collision of importance. They have never asked of the latter at any time editorial comment or commendation. Yet they deserve it. They have kept in Baltimore a Dental Depot, well supplying the wants of the profession in the South and quite largely in other parts of the country, and have been ever and always prompt, accomodating and reliable. We think all who have dealt with them will testify to this. They have been manufacturers, too, and some of the best things in use in the way of laboratory equipments, lathes, etc., have been invented and manufactured by them; but so quietly and with so little parade that it has passed partly unnoticed. Few know, perhaps, that the distinctive features of one of the newest and most popular dental chairs now sold, emanated from this house; and they made for years one of the best and most popular chairs ever devised, and one which is still largely used. So we feel, (although they do not ask for it,) like saying a good word for them, and acknowledging their services in the past and present in keeping up a Dental Journal in Baltimore at no profit to themselves.

